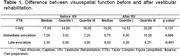# Improvement of visuospatial function in elderly people undergoing vestibular rehabilitation

**DOI:** 10.1002/alz70857_097517

**Published:** 2025-12-24

**Authors:** Marlon Bruno Nunes Ribeiro, Patricia Cotta Mancini, Maria Aparecida Camargos Bicalho

**Affiliations:** ^1^ Universidade Federal de Minas Gerais, Belo Horizonte, Minas Gerais, Brazil; ^2^ Universidade Federal de Minas Gerais, Belo Horizonte, Brazil; ^3^ Department of Clinical Medicine, Faculty of Medicine, Universidade Federal de Minas Gerais (UFMG), Belo Horizonte, Minas Gerais, Brazil

## Abstract

**Background:**

The visuospatial function has an excellent relationship with the vestibular system, being a more scientific cognitive ability. However, there are few studies that evaluated visuospatial function before and after vestibular rehabilitation, especially in the Brazilian literature. The purpouse is verify whether elderly people with vestibular dysfunction undergoing vestibular rehabilitation can show improvement in visuospatial function.

**Method:**

Longitudinal, analytical and quasi‐experimental study (interrupted time series). The sample consisted of 52 elderly people, of both sexes, aged between 60 and 86 years old. Elderly people with vestibular dysfunction confirmed by the Vestibular Evoked Myogenic Potential (VEMP) and Video Head impulse Test (v‐HIT) tests were included. Visuospatial function was assessed before and after eight vestibular rehabilitation (VR) sessions using the Taylor Complex Figure (simplified). The variables were compared before and after vestibular rehabilitation using the Wilcoxon test. However, for intra‐subject analysis before and after intervention, the Reliable Change Index (RCI) was used. A significance level of 5% (*p* < 0.05) was adopted.

**Result:**

Participants showed improvement in visuospatial function, especially in immediate recall and delayed recall tasks.

**Conclusion:**

After vestibular rehabilitation, the elderly showed better performance in visuospatial function.